# Borderline personality disorder and prior suicide attempts define a severity gradient among hospitalized adolescent suicide attempters

**DOI:** 10.1186/s12888-020-02930-4

**Published:** 2020-11-04

**Authors:** Aveline Aouidad, David Cohen, Bojan Mirkovic, Hugues Pellerin, Sébastien Garny de La Rivière, Angèle Consoli, Priscille Gérardin, Jean-Marc Guilé

**Affiliations:** 1 Hopital Universitaire Pitie Salpetriere, Paris, France; 2 Institut des Systemes Intelligents et de Robotique, Paris, France; 3 Hopital Charles Nicolle, Rouen, France; 4 Centre Hospitalier Universitaire Amiens-Picardie, Amiens, France

**Keywords:** Borderline personality disorder, Suicide attempt, Adolescents, Anxiety disorder, Non-suicidal self-injury, Inpatients

## Abstract

**Background:**

Borderline personality disorder (BPD) and history of prior suicide attempt (SA) have been shown to be high predictors for subsequent suicide. However, no previous study has examined how both factors interact to modify clinical and suicide severity among adolescents.

**Methods:**

This study presents a comprehensive assessment of 302 adolescents (265 girls, mean age = 14.7 years) hospitalized after a SA. To test clinical interactions between BPD and history of prior SA, the sample was divided into single attempters without BPD (non-BPD-SA, *N* = 80), single attempters with BPD (BPD-SA, *N* = 127) and multiple attempters with BPD (BPD-MA, *N* = 95).

**Results:**

Univariate analyses revealed a severity gradient among the 3 groups with an additive effect of BPD on the clinical and suicide severity already conferred by a history of SA. This gradient encompassed categorical (anxiety and conduct disorders and non-suicidal-self-injury [NSSI]) and dimensional comorbidities (substance use and depression severity) and suicide characteristics (age at first SA).

According to regression analyses, the BPD-MA group that was associated with the most severe clinical presentation also showed specific features: the first SA at a younger age and a higher prevalence of non-suicidal self-injury (NSSI) and anxiety disorders. The BPD-MA group was not associated with higher impulsivity or frequency of negative life events.

**Conclusions:**

Based on these findings and to improve youth suicide prevention, future studies should systematically consider BPD and the efficacy of reinforcing early interventions for anxiety disorders and NSSI.

**Supplementary Information:**

The online version contains supplementary material available at 10.1186/s12888-020-02930-4.

## Background

Suicide is the second leading cause of death among youth in the United States and most European countries and is responsible for up to 16% of deaths among 15- to 24-year-old individuals in Europe [1, 2]. Preventing the repetition of suicide attempts (SAs) among adolescents is a fundamental issue because it is a significant risk factor for future completed suicide [3, 4]. Many causes leading adolescents to attempt suicide have been studied, but their specific implications and relationships remain unclear. Identifying high-risk groups among self-harmers may help elucidate the interactions between factors leading adolescents to attempt suicide [5].

Borderline personality disorder (BPD) and history of suicidal behaviors have both independently been shown to be high predictors for subsequent SAs among adults and adolescents [3, 6–8]. However, no previous study has examined how both factors interact to modify the risk for repeated attempts among adolescents and whether other clinical or demographic characteristics could reduce or enhance the risks associated with these factors. Indeed, although several studies aimed to identify the risk factors for repeated suicide attempts in adults suffering from BPD, no studies in adolescents considered these two factors [9–13]. BPD is a severe mental illness characterized by emotional and relational instability, impulsivity, non-suicidal self-injury (NSSI) and suicidal behaviors, that begins in adolescence and affects up to 11–22% of adolescent outpatients [14–16]. It is associated with a very high lifetime risk for suicide, with a standardized mortality rate found to be 45 times higher than in general population [17].

Using a categorical approach, BPD is considered to confer an additional risk for SAs relative to major depressive disorder (MDD), both among adults and adolescents [7, 8, 18]. Nevertheless it is still unclear what dimensionally prevails in the association between BPD and SAs. Indeed, some of BPD core symptoms, are known independent risk factors for SAs [5, 19, 20]. For example, impulsivity has been considered a precipitating factor for SA among patients with BPD [21–23]. But it is not known whether it is impulsivity in itself or its association with other BPD dimensions that is more likely to lead to suicidal behaviors. Similarly, BPD patients are known to suffer from an enhanced emotional reactivity to stressful events that may provoke SAs [18, 24–27]. Studying BPD more precisely in adolescents may therefore be an important step to better delineate the risk of repeated SAs.

In addition, a history of suicidal behaviors during adolescence has been shown to independently increase the risk for both subsequent SA by almost 4-fold [3] and for suicide-related death by more than 22-fold [6]. Multiple attempters have also been shown to present distinctive clinical features that may justify additional prevention and treatment. They exhibit increased levels of depression and hopelessness, increased numbers of psychiatric comorbidities and lower levels of functioning than single attempters [3, 28–30]. However, only a few studies have focused on adolescent multiple attempters, and the available studies did not control for BPD diagnosis [3, 31, 32]. This could be partly explained by the fact that many clinicians and researchers were until recently reluctant to diagnose youth with BPD, considering it as a pediatric personality deviation reflective of developmental stages [33]. However, the capacity to reliably diagnose BPD in youth has recently increased, and the validity of BPD diagnoses in adolescents as young as 11 years old is now well accepted [16, 34–36].

Based on these findings, we divided a large community sample of adolescent suicide attempters into groups of interest according to BPD diagnosis and history of prior SA. To disentangle the heterogeneity of adolescent suicide attempters, we compared socio-demographics, suicidal behaviors, psychiatric comorbidities, psychopathological dimensions and negative life events across the groups.

The aims of this study were 1) to determine whether single and multiple suicide attempters with BPD differ clinically from attempters without BPD and 2) to determine which specific pattern of socio-demographics, clinical comorbidities and psychopathological dimensions characterizes multiple attempters.

To the best of our knowledge, this is the first study to compare adolescent single and multiple suicide attempters in regard to BPD diagnosis. The results of this study may help clinicians evaluate suicide potential and target prevention strategies in adolescent populations.

## Methods

### Participants

Participants were included in a French multisite study designed to better understand suicidal behaviors among adolescents [26, 37, 38]. From November 2010 to November 2015, 320 adolescents aged 11 to 17 years (83% girls, 17% boys) who were hospitalized after a SA were recruited from 5 inpatient units in France, in pediatric and child psychiatry departments (Rouen, Amiens, Crepy, Creil, and Meaux). The exclusion criteria included the following: inability to provide written informed consent (for example, moderate to severe cognitive impairment), acute medical conditions and residence outside of the geographical area of each center to limit patient loss to follow-up. Consent for minors was obtained from each adolescent as well as from both parents. The Nord-Ouest I Medical Ethics Committee of Rouen University Hospital approved the study.

### Procedure and measures

Baseline measures were obtained from self-reported questionnaires and face-to-face interviews performed by a research psychiatrist or resident. Socio-demographics, family and education variables and personal medical and psychiatric history were assessed by interview. Psychiatric comorbidities, psychopathology, negative life events and suicidal behaviors were collected for each participant using the scales described below.

The groups of interest were defined using 2 criteria: a) the history of prior SA according to the Columbia-Suicidal Severity Rating Scale (C-SSRS) [39, 40] with the multiple suicide attempters group defined by at least one prior SA; and b) the presence of a BPD diagnosis according to the Abbreviated-Diagnostic Interview for Borderlines (Ab-DIB) [41]. Participants were included in the BPD group if the Ab-DIB total score was above the clinical threshold and all available data confirmed the diagnosis according to the consensus best estimate procedure [42].

The Ab-DIB [41] is a self-report derived from the Diagnosis Interview for Borderline-Revised (DIB-R) [43], which was tested on 139 suicidal adolescents for reliability. Internal consistency and test-retest intraclass correlation coefficients ranged from 0.80 to 0.86 and 0.77 to 0.95, respectively. Concurrent validity was tested against the DIB-R. Receiver operating characteristic analysis yielded an area under the curve of 0.87 (*P* > 0.001), indicating good diagnostic accuracy. The sensitivity was 0.88, and the specificity ranged from 0.82 to 0.73 depending on the age range. Each item was scored from 0 to 2. A total score higher than 12 indicated BPD. The same categorization was previously used on another adolescent suicide attempter sample and demonstrated good stability at a 4-year follow-up. Seventy-six percent of suicidal youth at recruitment surpassing the cutoff criteria for BPD according to the Ab-DIB met those same criteria 4 years later [7].

The severity of suicidal ideations in the past month, actual attempt behavior severity, the number and characteristics of past attempts and NSSI frequency were assessed using the 10-min clinician-administered *Columbia-Suicidal Severity Rating Scale* (C-SSRS) (Posner et al. 2007). Continuous outcome variables were generated with the C-SSRS algorithm used in the TORDIA study [39]: 1) a rating of ‘suicidal ideation’ ranging from 0 to 5 (no ideation to suicidal ideation with intent and a clear plan) and 2) a rating of ‘suicidal behavior’ ranging from 0 to 5 (no behavior to multiple attempts during the assessment period). In addition, we computed a composite variable by averaging the two scores on a 0–5 rating scale labeled the ‘suicide severity’ rating. This scale has demonstrated good psychometric qualities (sensitivity 100%; specificity 99.4%; internal consistency [Cronbach alpha]: 0.73; convergent validity with several other instruments varies from 0.34 to 0.69, *P* <  0.001) [44].

Lifetime psychiatric comorbidities were explored using the well-established *Schedule for Mood Disorders and Schizophrenia for Children and Adolescents of School Age, Present and Lifetime Version* (K-SADS-PL) (Kaufman et al., 1997*).* This semi-structured interview designed to assess DSM-IV-R Axis I main diagnoses has shown good metric qualities at the diagnostic level (interrater reliability from 93 to 100%; test–retest: 0.74 to 0.90) [45]. Here, the variable labeled ‘anxiety disorder’ encompasses lifetime diagnoses of simple phobias, generalized anxiety disorder, panic disorder, agoraphobia, social phobia and post-traumatic stress disorder; the variable ‘ODD/CD’ encompasses lifetime diagnoses of oppositional defiant disorder (ODD) and conduct disorder (CD).

Several continuous variables of interest in regard to adolescents’ suicidal behaviors were assessed using the following scales as they have shown good psychometric properties: (i) the *Children Global Assessment Scale* (CGAS), which proposes a score ranging from 1 (extremely poor functioning) to 100 (highest functioning) (Schaffer et al., 1983); (ii) the *Dependence Questionnaire for Adolescents* (DEP-ADO), which is a self-report inventory used to assess substance use and misuse among adolescents (Landry et al. 2004). The DEP-ADO has been tested for reliability and validity in a sample of 673 adolescents from secondary schools in Quebec and demonstrated good psychometric qualities in regard to abusive consumption of psychoactive substances among adolescents [46]. Here, we used the total score. (iii) The *Beck Depression Inventory Second Edition* (BDI-II) is a self-report inventory used to assess depressive symptoms in the past 2 weeks. The BDI-II comprises 21 items rated on a 4-point scale from 0 (not present) to 3 (severe); the values are then summed for a total score ranging from 0 to 63 with higher scores reflecting a higher level of depression: 0 to 13, none or minimal; 14 to 19, mild; 20 to 28, moderate; and 29 to 63, severe (Beck et al., 1996, Bouvard et al., 2002). (iv) The *Beck Hopelessness Scale* (BHS) is a self-report inventory used to assess negative expectations regarding the future among adolescents and adults. The BHS comprises 20 true or false items distributed across 3 factors: feelings about the future, loss of motivation, and future expectations. The true responses are then summed for a total score ranging from 0 to 20, with higher scores reflecting higher levels of hopelessness: 0 to 3, normal; 4 to 8, mild; 9 to 14, moderate; and 15 to 20, severe (Beck et al., 1974). (v) The *Eysenck’s Impulsivity Inventory* for adolescents is a 23-item self-report inventory used to assess the personality traits of impulsivity among adolescents aged 7–18 years old [47]. (vi) The Relationship Scales Questionnaire (RSQ) is a 17-item self-report inventory [48] used to assess values of attachment styles: secure and insecure (fearful, preoccupied, dismissing). (vii) The *Newcomb Life Events Questionnaire for Adolescents* (Newcomb et al., 1981) is a 39-item self-report inventory used to examine the past-year negative life events of adolescents aged 14 to 18 years old. The scale is completed in 3 steps. First, respondents must rate how each event would make them feel on a 5-point scale from 1 (very unhappy) to 5 (very happy). Second, they are asked to indicate whether they experienced the events in the past year. Third, they are asked to indicate whether they experienced these same events more than 1 year earlier. Here, we defined a negative life event as one that occurred in the past year and that was rated as ‘unhappy’ or ‘very unhappy’.

A description of the variables is presented in the Additionnal file 1 (Supplementary Material).

### Statistical analyses

The population was divided into three groups: single attempters without BPD (non-BPD-SA), single attempters with BPD (BPD-SA) and multiple attempters with BPD (BPD-MA). Because the number of multiple attempters without BPD was limited to 7 participants (2.2% of the total sample), we decided to exclude them from the analyses. Sensitivity analyses were performed to address the validity of this decision. First, a univariate analysis was performed to describe and compare clinical features across the three groups. Given the non-parametric distribution of the continuous variables (graphically assessed), we performed Kruskal-Wallis tests to compare features across groups. Categorical variables were compared using either chi^2^ or Fisher’s exact tests. Given the exploratory nature of the study, we reported unadjusted *p*-values [49]. Second, a multinomial logistic regression was conducted to explain the group classification based on explicative clinical variables. Modeling was performed following these steps: for variable selection, we started by defining candidate variables in the sample and variables that should be forced into the model (sex and age at first attempt). Multicollinearity was avoided using domain knowledge and by inspection of the correlation matrix. We then calculated the maximum number of explanatory variables we could use before the occurrence of over-fitting using classical rules of thumb. If that number was reached, we used a stepwise algorithm to reduce the number of explanatory variables. After variable selection was performed, missing data were handled by multiple imputations in the dataset (non-parametric random forest method, using R missForest package). The input was composed of the selected variables, some auxiliary variables (chosen for their correlation with the outcome and their number of missing values) and the outcome. Multivariate modeling was finally performed on the imputed dataset with 17 selected explanatory variables. A *p*-value lower than 0.05 was considered significant. The statistical analyses were performed using R software 3.4.2.

## Results

### Participants

**Fig. 1:**
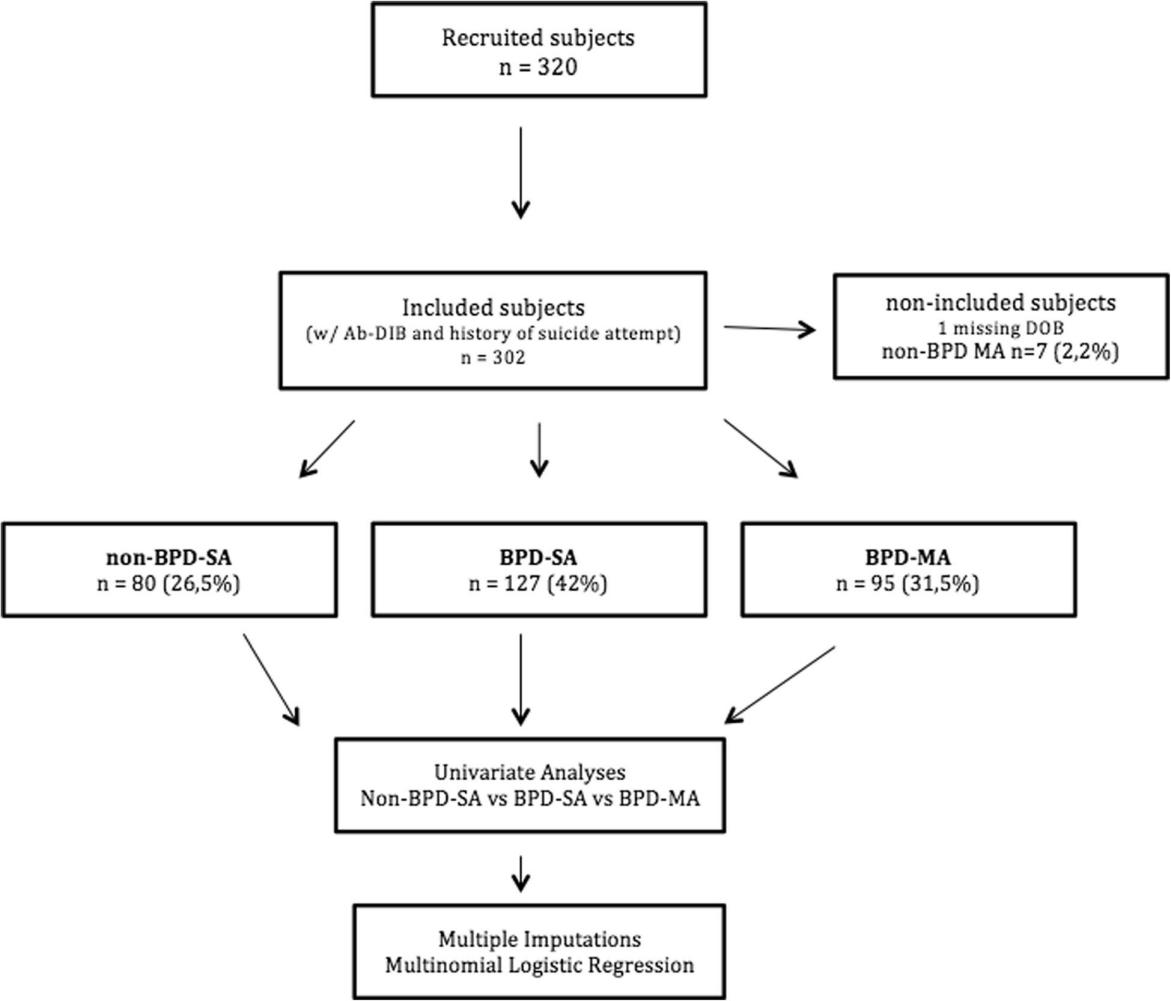
Flow chart. BPD-SA: single attempters with BPD. BPD-MA: multiple attempters with BPD. non-BPD-SA: single attempters without BPD. non-BPD-MA: multiple attempters without BPD. Ab-DIB: Abbreviated – Diagnostic Interview for Borderline

Three hundred and twenty adolescents hospitalized after a SA were included in this study (see diagram flow in Fig. 1). Three hundred thirteen participants had completed the Ab-DIB self-report [41] and information on the history of prior suicide attempts was available for 310 of these participants. Only seven multiple attempters did not meet the criteria for borderline personality disorder and were excluded from further analysis. Date of birth was missing for one subject. Thus, we included 302 participants in the analyses: 80 (26.5%) non-BPD-SA, 127 (42%) BPD-SA and 95 (31.2%) BPD-MA. The socio-demographic characteristics of the population are reported in Table 1. The whole sample was predominantly female, with 265 (83%) girls and 54 (17%) boys. The mean age was 14.7 years (±1.29, range: 11.5–17.7), the mean level of functioning based on the CGAS was 68.8 (±15.47, range: 5–95) and the mean level of depression based on the BDI-II was 25.28 (±13.9, range: 0–62). One of the centers (Crepy, *n* = 14) was overrepresented in the third BPD-MA group. The three groups did not differ in terms of age at admission or gender. The groups also did not differ in terms of parental level of education or domiciliation, with a majority of adolescents living without both parents. The mean number of suicide attempts of multi-attempters was 2.8 (±1.27).

**Table 1:** Sociodemographics and clinical characteristics of groups (*n* = 302)

Groups	Non-BPD-SA *N* = 80(26.5%)	BPD-SA *N* = 127(42%)	BPD-MA *N* = 95(31,5%)	*p*-value
*Sociodemographics*				
Mean Age (SD, min, max) (*N* = 297)	14.71(1.47)	14.59(1.17)	14.89(1.3)	0.3
Sex, Female n (%): Men n (%) (*N* = 301)	61(76.2%): 19(23.8%)	109(86.5%): 17(13.5%)	80(84.2%): 15(15.8%)	0.15
Site of inclusion (n, %) (*N* = 302)				–
Rouen, Amiens, Meaux, Creil, Crépy	44, 19, 4, 4, 6	75, 40, 3, 4, 4	50, 27, 4, 1, 14	0.027*
*Family Variables*				
Domiciliation: w/o or w/ both parents, n(%) (*N =* 301)	42(52.5%) / 38(47.5%)	80(63%) / 47(37%)	56(59.6%) / 38(40.4%)	0.325
Parental level of education^b^				–
Mother, mean (SD) (*N* = 173)	2.93(1.13)	2.93(1.14)	2.85(1.22)	0.871
Father, mean (SD) (*N* = 140)	2.85(1.35)	2.93(1.24)	2.77(1.25)	0.762
Number of siblings, mean (SD) (*N* = 298)	2.53(1.38)	2.46(1.26)	2.47(1.3)	0.962
Rank among siblings	1.57(1.16)	1.67(1.23)	1.73(1.3)	0.76
*Education Variables*				
Special class services n(%) (yes) (*N* = 294)	67(85.9%)	108(88.5%)	81(86.2%)	0.822
Grade repetition n(%) (yes) (*N* = 299)	32(40.5%)	27(21.3%)	36(38.7%)	0.003**
*Attachement Style (RSQ)*				
Insecure Attachement Style, mean (SD) (*N* = 259)	9.63(3.5)	11.96(3.35)	13.29(3.39)	< 0.001***
*Negative Life Events*				
Familial, parental (*N* = 290)	0.65(1.07)	0.72(1.28)	0.56(0.75)	0.982
Accident, illness (*N* = 274)	0.61(0.8)	0.69(0.84)	0.65(0.82)	0.807
Sexual (*N* = 274)	0.37(0.59)	0.61(0.73)	0.56(0.68)	0.056
Autonomy (*N =* 274)	0.01(0.12)	0.07(0.26)	0.08(0.27)	0.181
Deviance (*N =* 274)	0.24(0.58)	0.15(0.36)	0.23(0.45)	0.414
Relocation (*N =* 274)	0.1(0.35)	0.27(0.48)	0.26(0.51)	0.022*
Distress (*N =* 274)	1.2(1.11)	1.38(1)	1.11(0.98)	0.152
Other (*N =* 274)	0.04(0.21)	0.06(0.24)	0.08(0.27)	0.635
Total (*N =* 274)	3(2.41)	3.76(2.31)	3.51(2.21)	0.057
*Medical and Psychological Care*				
Psychiatric care n(%) (yes) (*N =* 253)	21(31.8%)	30(27.8%)	45(57%)	< 0.001***
*Medication* (*N =* 302)				
Antidepressant n(%) (yes)	1(1.2%)	7(5.5%)	14(14.7%)	0.002**
Anxiolytic n(%) (yes)	6(7.5%)	17(13.4%)	14(14.7%)	0.305
Antipsychotic n(%) (yes)	2(2.5%)	2(1.6%)	12(12.6%)	0.001**
Thymoregulator n(%) (yes)	1(1.2%)	0(0%)	3(3.2%)	0.131
*Clinical Characteristics (Categorical and Dimensional)*				
*Psychiatric diagnoses (Lifetime, K-SADS)*				
Major Depressive Disorder, n(%) (yes) (*N* = 291)	22(28.2%)	50(40.3%)	58(65.2%)	< 0.001**
Anxiety Disorder, n(%) (yes) (*N =* 291)	13(16.9%)	32(25.6%)	41(46.1%)	< 0.001***
ADHD, n(%) (yes) (*N =* 291)	3 (3.9%)	4 (3.2%)	10 (11.2%)	0.044*
ODD and/or Conduct Dis., n(%) (yes) (*N =* 291)	6(7.8%)	29(23.2%)	28(31.5%)	0.001**
Eating Disorder, n(%) (yes) (*N* = 292)	2(2.6%)	1(0.8%)	4(4.5%)	0.21
Substance use (DEP-ADO), mean (SD) (*N =* 274)	3.74(3.81)	7.37(6.93)	10.18(8.61)	< 0.001***
*Psychopathology*				
Level of depression (BDI), mean (SD) (*N =* 297)	12.83(9.05)	27.6(11.86)	32.97(13.09)	< 0.001***
Level of hopelessness (BHS), mean (SD) (*N =* 291)	6.03(4.5)	9.8(5.25)	11.74(5.53)	< 0.001***
Impulsivity (Eysenck scale), mean (SD)(*N* = 284)	9.64(4.34)	12.83(4.57)	13.66(4.6)	< 0.001***
CGAS, mean (SD) (*N* = 271)	73.86(13.54)	71.65(15.02)	61.45(15.13)	< 0.001***
*Suicidal Assessment (Actual, C-SSRS)*				
Age at first attempt, mean (SD) (*N* = 251)	14.73(1.48)	14.59(1.18)	14.05(1.59)	0.017*
Suicide severity, mean (SD) (*N* = 262)	1.76(2.05)	3.29(2.22)	4.17(1.93)	< 0.001***
Non-Suicidal Self-Injury (NSSI) n(%) (yes) (*N =* 284)	18(23.4%)	49(41.5%)	62(69.7%)	< 0.001***

* *p* < .05, ** *p* < .01, *** *p* < 0.001

*ODD* Oppositional Defiant Disorder, *ADHD* Attention Deficit Hyperactivity Disorder, *RSQ* Relationship Questionnaire, *DEP-ADO* Dependency Scale for Adolescents, *BDI* Beck Depression Inventory, *BHS* Beck Hopelessness Scale, *C-SSRS* Columbia–Suicide Severity Rating Scale, *CGAS* Children Global Assessment Scale

### Univariate analysis

Nineteen variables significantly differed between the non-BPD-SA, BPD-SA and BPD-MA groups. The results from the univariate analysis are reported in Table 1. For all these univariate differences, we found a severity gradient considering each variable across the 3 groups, with the non-BPD-SA group being the least severe and the BPD-MA group being the most severe. Three clinical comorbidities were significantly different in prevalence between the groups: major depressive disorder (MDD), ODD/CD and anxiety disorder. Three suicidal characteristics were different between the three groups: age at first attempt, suicide severity and NSSI frequency. Six dimensional variables were different between the three groups: depression severity and hopelessness, impulsivity, substance use, global level of functioning and insecure attachment style. Among the factors related to medical and psychiatric history, three were different: personal psychiatric history, antidepressant medication use and antipsychotic medication use. Finally, one educational variable and one negative life event were different across the three groups: grade repetition and relocation in the past year. Of note, the site of inclusion was different across the three clinical groups, with significantly more multiple attempters in the Crepy center.

### Multivariate analysis

From the univariate analysis, 24 variables had a *p*-value < 0.2 (see Table 1): the nineteen variables cited in the univariate analysis results section, gender and four negative life event variables (related to sexual factors, autonomy, distress events and total score). We found collinearity between hopelessness and depression severity. Because depression was a more robust and documented dimension, we decided to discard hopelessness from the model. The total negative life events score was also discarded for collinearity.

Regarding possible biases related to recruitment sites, we did not include the center in the multivariate analyses. Indeed, as indicated in the previous section, BPD-MA patients were overrepresented in the Crepy site. To assess whether this site including a small number of subjects (*N* = 25) changed our global models, we conducted a sensitivity analysis considering only the patients from the two main centers of Amiens and Rouen (*N* = 277). The models did not differ (Supplementary Material). Additionally, we forced family variables in the multivariate analysis: domiciliation and parental level of education as adjustment factors because they are known to influence suicidal behaviors. A stepwise variable selection in both directions was then conducted with the 24 selected variables, leading to a multivariate model with 17 variables based on AIC statistics.

Multinomial logistic regression was performed to examine how these 17 sociodemographic and clinical variables predict the classification of the 3 groups of interest: non-BPD-SA, BPD-SA and BPD-MA. The model was significant, and the unique effect of all predictors is presented in Table 2 with their corresponding odds ratios [ORs], 95% confidence intervals [CIs] and *p*-values.

- BPD versus non-BPD single attempters (BPD-SA vs. non-BPD-SA)

**Table 2:** Logistic multinomial regressions comparing groups

	BPD SA vs Non BPD SA				BPD MA vs Non-BPD SA				BPD MA vs BPD SA			
	Adjusted odds ratio	(95%IC)		*p-*value	Adjusted odds ratio	(95%IC)		*p-*value	Adjusted odds ratio	(95%IC)		*p-*value
*Sociodemographics*												
Male gender	0.37	0.14	1.01	0.053	0.47	0.15	1.52	0.210	1.26	0.53	3.01	0.597
Mother’s education level	0.72	0.45	1.14	0.163	0.68	0.40	1.14	0.141	0.94	0.66	1.32	0.717
Domiciliation (w/ both parents)	0.81	0.36	1.82	0.615	1.11	0.43	2.82	0.832	1.36	0.72	2.59	0.347
*Negative Life events (LEQ) (past year)*												
Sexual	1.54	0.75	3.16	0.239	1.38	0.62	3.08	0.432	0.90	0.55	1.46	0.658
Autonomy	1.85	0.15	22.74	0.632	1.12	0.08	15.60	0.931	0.61	0.19	2.00	0.413
Relocation	2.15	0.74	6.29	0.161	2.83	0.87	9.14	0.082	1.31	0.68	2.52	0.413
*Clinical Characteristics*												
*Psychiatric comorbidities (past and present)*												
Major Depressive Disorder	0.60	0.24	1.50	0.276	0.84	0.30	2.31	0.733	1.39	0.72	2.67	0.324
**Anxiety Disorder**	1.98	0.72	5.44	0.188	**3.75**	**1.26**	**11.19**	0.018*	1.90	0.97	3.72	0.062
**ODD/CD**	**4.84**	**1.24**	**18.87**	0.023*	3.68	0.83	16.20	0.085	0.76	0.34	1.69	0.498
ADHD	0.49	0.07	3.57	0.481	1.13	0.13	10.25	0.912	2.31	0.50	10.78	0.286
*Psychopathology (past and present)*												
**Level of depression (BDI)**	**1.18**	**1.11**	**1.24**	< .001***	**1.19**	**1.12**	**1.26**	< .001***	1.01	0.98	1.04	0.395
Level of Impulsivity (Eysenck scale)	1.07	0.98	1.18	0.138	1.06	0.95	1.18	0.299	0.99	0.91	1.07	0.768
**Level of functioning (CGAS)**	**1.04**	**1.01**	**1.08**	0.012*	1.01	0.97	1.05	0.647	**0.97**	**0.94**	**0.99**	0.006**
**Substance use (DEP-ADO)**	**1.12**	**1.02**	**1.23**	0.019*	**1.16**	**1.05**	**1.28**	0.004**	1.03	0.98	1.09	0.176
*Suicidal Assessment (present)*												
**Age at first attempt**	0.90	0.66	1.23	0.517	**0.63**	**0.44**	**0.92**	**0.015**	**0.70**	**0.53**	**0.53**	0.013*
**Suicidal severity (C-SSRS)**	**1.54**	**1.23**	**1.91**	< .001***	**1.75**	**1.37**	**2.25**	< .001***	1.14	0.97	1.35	0.111
**Non-Suicidal Self-Injury (NSSI)**	1.36	0.54	3.44	0.510	**3.91**	**1.43**	**10.71**	0.008**	**2.87**	**1.53**	**5.37**	0.001**

* *p* < .05, ** *p* < .01, *** *p <*  0.001 (*p-*values are unajusted)

*ODD* Oppositional Defiant Disorder, *ADHD* Attention Deficit Hyperactivity Disorder, *RSQ* Relationship Questionnaire, *DEP-ADO* Dependency Scale for Adolescents, *BDI* Beck Depression Inventory, *BHS* Beck Hopelessness Scale, *C-SSRS* Columbia–Suicide Severity Rating Scale, *CGAS* Children Global Assessment Scale

Five clinical variables were associated with a significant OR when considering BPD-SA versus non-BPD-SA as a reference level: one categorical variable and four dimensional variables. Lifetime ODD/CD conferred an almost five-times higher odds of being part of the BPD-SA group ([OR] 4.84, [CI] 1.24–18.87; *p* = 0.023). A higher level of suicide severity (*P* <  0.001), increased severity of substance use (*P* = 0.019) and increased level of functioning (*P* = 0.012) were significantly associated with the BPD-SA group. Although no association was found for lifetime MDD, a 1-point increase in depressive symptoms on the BDI-II was associated with 1.18-fold higher odds of being part of the BPD-SA group (*P* <  0.001). Finally, female gender tended to be associated with BPD-SA (*P* = 0.053).

- BPD multiple attempters versus non-BPD single attempters (BPD-MA vs. non-BPD-SA)

Six clinical variables were associated with a significant OR when considering BPD-MA versus non-BPD-SA as a reference level: two categorical variables and four dimensional variables. Lifetime anxiety disorder conferred almost four-times higher odds of being part of the BPD-MA group ([OR] 3.75, [CI] 1.26–11.19; *p* = 0.018). Lifetime ODD/CD also tended to be associated with the BPD-MA group ([OR] 3.68, [CI] 0.83–16.20; *p* = 0.085). NSSI conferred almost four-times higher odds of being part of the BPD-MA group ([OR] 3.91, [CI] 1.43–10.71; *p* = 0.008). Higher levels of suicide severity (*P* <  0.001) and increased severity of substance use (*P* = 0.004) were significantly associated with the BPD-MA group. Although no association was found for lifetime MDD, a 1-point increase in depressive symptoms on the BDI-II was associated with 1.19-fold higher odds of being part of the BPD-MA group (*P* <  0.001). Finally, individuals in the BPD-MA group were significantly younger at their first SA (*P* = 0.015).

- BPD multiple attempters versus BPD single attempters (BPD-MA vs. BPD-SA)

Three clinical variables were associated with a significant OR when considering BPD-MA versus non-BPD-SA as a reference level: one categorical variable and two dimensional variables. NSSI conferred almost three-times higher odds of being part of the BPD-MA group ([OR] 2.87, [CI] 1.53–5.37; *P* = 0.001). Lifetime anxiety disorder also tended to be associated with the BPD-MA group ([OR] 1.90, [CI] 0.97–3.72; *P* = 0.062). A decreased level of functioning (*P* = 0.006) was significantly associated with the BPD-MA group. Finally, individuals in the BPD-MA group were significantly younger at their first SA (*P* = 0.013).

Notably, there were no significant associations between groups and family variables or negative life events.

## Discussion

The present study aimed to describe adolescent suicide attempters and identify at-risk groups while considering BPD diagnosis and history of SAs. The sample encompassed three groups: single attempters without BPD (non-BPD-SA) (*n* = 80, 26.5%), single attempters with BPD (BPD-SA) (*n* = 127, 42%) and multiple attempters with BPD (BPD-MA) (*n* = 95, 31.5%). Important differences in the clinical profiles of these groups emerged, confirming the relevance of this categorization of adolescent suicide attempters.

First, we found a severity gradient among the 3 groups with an additive effect of BPD on clinical and suicide severity already conferred by a history of SA (non-BPD-SA < BPD-SA < BPD-MA). The univariate analyses elicited a severity gradient covering categorical and dimensional comorbidities, suicide characteristics and the level of functioning. The multivariate analyses then allowed us to specify which variables provided an independent contribution to this gradient. Notably, young age at first attempt, NSSI and anxiety disorders were associated with the highest degree of clinical severity, and NSSI conferred almost 3-times higher odds of belonging to the BPD-MA group.

Second, our results confirm that adolescent suicide attempters are at a high risk of suffering from BPD. Indeed, in this inpatient sample, the prevalence of BPD diagnosis was 73.5% versus 44.7% for MDD, highlighting the high frequency of BPD diagnoses among adolescent suicide attempters. Greenfield and colleagues, applying a similar design in a cohort of 286 suicidal adolescents admitted to an emergency department, found an even higher rate of 87.7% of adolescents meeting the criteria for BPD [7]. Also, the very limited number of non-BPD-MA (*N* = 7) in this sample is an interesting result as it seems to indicate that in this young age range, BPD is highly associated with multiple suicide attempts. BPD diagnoses should therefore be more consistently investigated in studies related to adolescent suicide. More precisely, these results support the relevance of the systematic assessment of BPD in adolescents admitted to the emergency department after a SA [50].

Third, consistent with the literature, the results confirm that BPD in adolescent suicide attempters is highly comorbid with both externalizing and internalizing disorders [51, 52]. Confirming previous reports [7, 52], both BPD groups presented an increased level of substance abuse, as assessed with a dimensional scale. Among single attempters, history of ODD/CD leads to an almost five-times higher odds of belonging to the BPD group. However, unlike the results of other studies [53, 54], ADHD diagnosis was not associated with BPD diagnosis. This negative result might be due to the low frequency of ADHD in our sample, which weakened the statistical power. The severity of depressive symptoms, as assessed with the BDI-II, was significantly associated with BPD diagnosis. In contrast, despite a higher frequency of MDD, as assessed with the K-SADS-PL in the two BPD sub-groups, MDD did not provide an independent contribution to the sub-grouping of the sample. This might result from differences between self- and hetero-assessment of depressive symptoms. Indeed, young age and female gender are associated with higher scores of self-reported depressive symptoms [37, 55, 56]. This discrepancy might also result from differences in construct and assessment methods: dimensional severity assessed by the BDI-II self-questionnaire and categorical diagnosis assessed by the semi-structured interview K-SDAS-PL. Because a high proportion of adolescent suicide attempters suffered from BPD in this sample, a dimensional assessment of depressive symptoms may be more appropriate. Indeed, BPD patients present an emotional dysregulation characterized by transient depressive symptoms [57, 58] that can be referred to as intrapsychic pain [59]. Although they are transient, these severe symptoms are known to precipitate suicidal behaviors. However, these patients might not fulfill the criteria of an MDD diagnosis in terms of symptom duration. Therefore, as assessed in previous reports [7, 37], in adolescents hospitalized for SAs, it is more the intensity of depressive symptoms than the history of MDD that seems relevant for the prediction of suicidal risk. Overall, these results support the relevance of both dimensional and categorical assessments for the evaluation of adolescent suicide attempters [60].

Fourth, the BPD-MA group was associated with a younger age at first attempt and a specific pattern of externalizing and internalizing characteristics: NSSI and anxiety disorders. Significantly, NSSI and anxiety disorders independently conferred almost four-times higher odds of being part of the BPD-MA group. Indeed, although NSSI is one of the diagnostic criteria for BPD, its frequency allows to discriminate single from multiple attempters within BPD adolescents. These results confirm that NSSI and childhood anxiety disorders are important predictors of multiple attempts and are strongly associated with subsequent BPD diagnosis [3, 61–64]. This pattern of severe symptoms may arise from particular underlying pathophysiological mechanisms resulting from developmental or historical factors, as suggested by other authors [3, 65, 66]. Indeed, NSSI and early onset of suicidal behaviors have been shown to predict the onset of later mental illness, such as BPD, depression, anxiety disorders and substance abuse [20, 28, 29, 67–70]. Properly screening for and treating anxiety and NSSI should be encouraged, especially at an early age when BPD may not yet be possible to identify. Anxiety disorders and NSSI could be considered the earliest emerging symptoms among those who later engage in multiple suicide attempts. Based on these findings, adolescents with early onset of suicidal behaviors (i.e., ≤ 14 years old), anxiety disorder and NSSI would benefit the most from intensive interventions. Furthermore, even though it was discarded from the model after stepwise selection, insecure attachment style based on the RSQ was significantly more prevalent among BPD adolescents and specifically among BPD-MA individuals. This could support the hypothesis that the level of insecure attachment style is a mediator between BPD and self-injury [71].

Finally, in this sample of severe suicide attempters, impulsivity and negative life events did not predict group membership. Impulsivity level was not associated with any group, although it has been previously related to both multi-attempters and to BPD diagnosis [7, 72]. This finding goes against the generally accepted statement that impulsivity mediates the relationship between BPD and suicide attempts [12, 23]. This could support the hypothesis that impulsivity, by promoting exposure to stressors, would rather act as a potentiator of anxiety [72, 73]. The lack of a difference in the number of negative life events between groups might lend support to the fact that chronic stress and daily hassle seem to be more predictive of suicide severity than acute stress or events [65, 66, 74]. Furthermore, Oquendo and colleagues in their large prospective longitudinal study found life events to be protective against suicidal behavior among BPD patients [59]. They therefore hypothesized that facing significant life events might help BPD patients to paradoxically cope better [59], as they would be forced to “organize” around a life event.

### Strengths and limitations

To the best of our knowledge, this is the first study to compare both history of suicide attempts and BPD among adolescent suicide attempters. The study was based on a large sample size of young adolescent inpatients and proposes a new categorization of adolescent suicide attempters. However, the present findings must be viewed in light of some limitations.

First, it is important to recall that this sample only included inpatient adolescents with a recent suicide attempt and who agreed to participate, perhaps limiting generalizability. Moreover, girls represent 83% of this sample of adolescent suicide attempters. Although the rate of deliberate self-harm has consistently been found to be higher among females, values rarely reach such a high disparity [1]. This might be because most studies are population-based rather than based on hospitalized patients. Second, the cross-sectional design of the study does not allow any predictive interpretation of the findings. Future studies should focus on prospective evaluations. Furthermore, the exploratory design of the study does not allow any explanatory conclusion. Third, considering the different groups, it is important to note that because of the limited size of the non-BPD multi-attempters group (*N* = 7), we were not able to fully address the question of the interactions between BPD and the number of suicide attempts. Future studies should aim to replicate these results in a larger sample that would allow for the examination of this specific population and the delineation of the separate effect of each factor. Additionally, as discussed by other authors [28], it is noteworthy that the categorization of multiple versus single attempters represents a single time point. Therefore, an unknown number of single attempters will become multiple attempters, thus potentially modifying some of the results. Also, about the overrepresentation of multiple attempters in one of the recruitment sites it can be related to the lack of inpatients beds in some regions that might lead to the selection of more severe patients. Here, a sensitivity analysis based on the two main recruitment sites confirmed the results, therefore ruling out a possible site effect. Fourth, negative findings should be interpreted with caution, taking into account that it is a severely affected population, which certainly decreases differences among groups and limits the significance of the results. Cumulative or chronic stress rather than discrete life events could be more relevant among adolescent suicide attempters [25, 65, 66]. It is also important to consider that self-assessment can lead to potential recall bias or underrepresentation concerning both negative life events and clinical symptoms. Furthermore, in BPD patients the distorted cognitions and affective instability might alter the reporting of symptoms. Report by an informant from the close environment of a BPD patient additionaly to a self-report could be a way to define more accurately this population [75, 76].

## Conclusion

Screening for BPD and prior suicide attempts seem to allow for the deciphering of the heterogeneity of adolescent suicide attempters and the identification of at-risk groups. We therefore propose a simple method to categorize adolescent suicide attempters admitted to the emergency department: screening for BPD and history of prior suicide attempts. The findings confirm the close relationship between BPD, the intensity of depressive symptoms and repeated suicide attempts among adolescents. Significantly, the analyses revealed a severity gradient characterized by an additive effect of BPD on the clinical and suicide severity already conferred by a history of SA. Furthermore, the results allowed for the identification of a specific pattern characterizing high-risk adolescents: early onset of suicidal behaviors (i.e., ≤ 14 years old), history of anxiety disorder and NSSI. To improve youth suicide prevention, it will be important that further research continues to investigate BPD among suicidal adolescents. Future studies might explore the efficacy of reinforcing early interventions on anxiety disorder and NSSI in preventing adolescent suicide risk.

### Aknowledgments

We thank all participants and research collaborators for their contribution to the study. This study was funded in part by the Rouvray Hospital and the Rouen University Hospital and by 2 sponsors: Fondation Pfizer and Fondation de France. None of the sponsors interfered with the study design, the analysis and interpretation of data or the writing of the draft.

## Supplementary Information

**Additional file 1:.** Description of variables.

**Additional file 2.**

## Data Availability

The datasets analysed during the current study are available from the corresponding author on reasonable request.

## Abbreviations

Ab-DIB: Abbreviated version of the diagnostic Interview for Borderline – Revised
BDI-II: Beck Depression Inventory Second Edition
BHS: Beck Hopelessness Scale
BPD: Borderline Personality Disorder
BPD-MA: Multiple attempters with BPD
BPD-SA: Single attempters with BPD
CGAS: Children Global Assessment Scale
C-SSRS: Columbia-Suicidal Severity Rating Scale
DEP-ADO: Dependence Questionnaire for Adolescents
K-SADS-PL: Schedule for Affective Disorders and Schizophrenia for School-Age Children-Present and Lifetime Version
MDD: Major Depressive Disorder
Non-BPD-SA: Single attempters without BPD
NSSI: Non-Suicidal Self-Injury
RSQ: Relationship Scales Questionnaire
