# Supplementary material for: Borderline personality disorder and prior suicide attempts define a severity gradient among hospitalized adolescent suicide attempters
Source: BMC Psychiatry. 2020 Nov 4;20:525. doi: 10.1186/s12888-020-02930-4 (PMC7643473; doi:10.1186/s12888-020-02930-4)
Supplement: Supplementary file 1 — Additional file 1:. Description of variables. [file 12888_2020_2930_MOESM1_ESM.docx]

Supplementary Material : Description of variables

| Domain | Measure | Type of variable |
| --- | --- | --- |
| Borderline Personality Disorder | Abbreviated version of the diagnostic Interview for Borderline – Revised | Categorical (yes/no) |
| History of prior suicide attempt | Interview assessment |  |
| *Sociodemographics* | Mean Age at admission | Continuous |
|  | Sex | Categorical (M/F) |
| Family Variables | Domiciliation | Categorical (yes/no) |
|  | Parental level of education | Continuous |
|  | Number of siblings, mean (SD) | Continuous |
| Education Variables | Special class services | Categorical (yes/no) |
|  | Grade repetition | Categorical (yes/no) |
| *Negative Life Events* | Newcomb Life Events Questionnaire for Adolescents, assessing the negative life events occurring in the last year (rated as “bad” or “really bad”) |  |
| *Suicidal Assessment* |  |  |
| Mean number of suicide atempt(s) | Interview assessment | Continuous |
| Mean Age at first attempt | Interview assessment | Continuous |
| Suicidal ideation severity | Columbia-Suicidal Severity Rating Scale (C-SSRS) | Continuous |
|  | Suicidal behavior severity (C-SSRS ), mean (SD) |  |
| *Psychiatric comorbidities* | DSM-IV Axis I main diagnoses by Standardized interview with the Schedule for Mood Disorders and Schizophrenia for Children and Adolescents of School Age, Version Current and Past Episode (Kiddie-SADS Present and Lifetime Version interview); | Categorical (yes/no) |
| Major Depressive Disorder (MDD) |  | Categorical (yes/no) |
| ODD and/or Conduct Disorder |  | Categorical (yes/no) |
| Eating Disorder |  | Categorical (yes/no) |
| ADHD |  | Categorical (yes/no) |
| Anxiety | Presence of at least one of following lifetime diagnosis:  Post Traumatic Stress Disorder, Generalized Anxiety Disorder, Social Phobia, other phobias and Obsessive Compulsive Disorder. | Categorical (yes/no) |
| Substance Abuse | Dependence Questionnaire for Adolescents (DEP-ADO) Total | Continuous |
| Non-Suicidal Self-Injury (NSSI) | Columbia-Suicidal Severity Rating Scale (C-SSRS) | Categorical (yes/no) |
| *Psychopathology* |  |  |
| Depression severity | Beck Depression inventory (BDI) | Continuous |
| Impulsivity | Total Eysenck scale for impulsiveness | Continuous |
| Self-esteem | Rosenberg Self-Esteem Rating Questionnaire | Continuous |
| Hopelessness | Beck Hopelessness Scale (BHS) | Continuous |
| *Attachement Style* | Relationship Style Questionnaire (RSQ) | Continuous |
| Functioning | Children Global Assessment Scale (CGAS) | Continuous |
| *Medical and Psychological Care* |  |  |
| Psychiatric care | Interview | Categorical (yes/no) |
| Psychotherapy | Interview | Categorical (yes/no) |
| Current Medication | Type of chemotherapy | Categorical (yes/no) |
