# Supplementary material for: Borderline personality disorder and prior suicide attempts define a severity gradient among hospitalized adolescent suicide attempters
Source: BMC Psychiatry. 2020 Nov 4;20:525. doi: 10.1186/s12888-020-02930-4 (PMC7643473; doi:10.1186/s12888-020-02930-4)
Supplement: Supplementary file 2 — Additional file 2. [file 12888_2020_2930_MOESM2_ESM.docx]

|  | BPD SA vs Non BPD SA | | BPD MA vs Non-BPD SA | | BPD MA vs BPD SA | |
| --- | --- | --- | --- | --- | --- | --- |
|  | Adjusted odds ratio | p-value | Adjusted odds ratio | p-value | Adjusted odds ratio | p-value |
| *Sociodemographics* |  |  |  |  |  |  |
| Male gender | **0.26** | **0.014*** | 0.34 | 0.099 | 1.34 | 0.553 |
| Mother's education level | 0.68 | 0.171 | 0.69 | 0.244 | 1.02 | 0.926 |
| Domiciliation (w/ both parents) | 0.92 | 0.845 | 1.34 | 0.567 | 1.46 | 0.285 |
| *Negative Life events (LEQ) (past year)* |  |  |  |  |  |  |
| Sexual | 1.81 | 0.114 | 1.77 | 0.178 | 0.98 | 0.929 |
| Autonomy | 1.72 | 0.662 | 1.34 | 0.822 | 0.78 | 0.688 |
| Relocation | 1.91 | 0.237 | 2.71 | 0.103 | 1.42 | 0.328 |
| *Clinical Characteristics* |  |  |  |  |  |  |
| *Psychiatric comorbidities (past and present)* |  |  |  |  |  |  |
| Major Depressive Disorder | 0.77 | 0.593 | 1.10 | 0.859 | 1.43 | 0.317 |
| **Anxiety Disorder** | 1.31 | 0.626 | 2.88 | 0.076 | **2.19** | **0.032*** |
| **ODD/CD** | **4.70** | **0.031*** | 3.96 | 0.081 | 0.84 | 0.700 |
| ADHD | 0.61 | 0.627 | 2.88 | 0.076 | 2.52 | 0.238 |
| *Psychopathology (past and present)* |  |  |  |  |  |  |
| **Level of depression (BDI)** | **1.17** | **< .001***** | **1.18** | **< .001***** | 1.02 | 0.285 |
| Level of Impulsivity (Eysenck scale) | 1.05 | 0.314 | 1.04 | 0.533 | 0.99 | 0.764 |
| **Level of functioning (CGAS)** | **1.05** | **0.011*** | 1.01 | 0.479 | **0.97** | **0.019*** |
| **Substance use (DEP-ADO)** | **1.12** | **0.027*** | **1.14** | **0.015*** | 1.02 | 0.465 |
| *Suicidal Assessment (present)* |  |  |  |  |  |  |
| **Age at first attempt** | 0.94 | 0.713 | **0.65** | **0.044*** | **0.70** | **0.021*** |
| **Suicidal severity (C-SSRS )** | **1.57** | **< .001***** | **1.68** | **< .001***** | 1.07 | 0.454 |
| **Non-Suicidal Self-Injury (NSSI)** | 1.41 | 0.496 | **4.18** | **0.010**** | **2.97** | **0.002**** |

* p < .05, ** p < .01, *** p < 0.001

ODD : Oppositional Defiant Disorder ; ADHD : Attention Deficit Hyperactivity Disorder ; RSQ : Relationship Questionnaire ; DEP-ADO : Dependency Scale for Adolescents ; BDI : Beck Depression Inventory ;

BHS : Beck Hopelessness Scale ; C-SSRS : Columbia–Suicide Severity Rating Scale ; CGAS : Children Global Assessment Scale

**Sensitivy Analysis.** Logistic multinomial regression predicting group membership based on the two main sites (N=277)
